# Optical and Thermal Investigations of New Schiff Base/Ester Systems in Pure and Mixed States

**DOI:** 10.3390/polym13111687

**Published:** 2021-05-22

**Authors:** Abeer S. Altowyan, Hoda A. Ahmed, Sobhi M. Gomha, Ayman M. Mostafa

**Affiliations:** 1 Department of Physics, College of Science, Princess Nourah bint Abdulrahman University, Riyadh 11671, Saudi Arabia; asaltowyan@pnu.edu.sa; 2 Department of Chemistry, Faculty of Science, Cairo University, Cairo 12613, Egypt; sm.gomha@iu.edu.sa; 3 Chemistry Department, College of Sciences, Yanbu, Taibah University, Yanbu 30799, Saudi Arabia; 4 Chemistry Department, Faculty of Science, Islamic University in Almadinah Almonawara, Almadinah Almonawara 42351, Saudi Arabia; 5 National Research Centre, Spectroscopy Department, Physics Division, El-Buhouth St., Dokki, Giza 12622, Egypt; aymanmdarwish@gmail.com; 6 Laser Technology Unit, Centre of Excellence for Advanced Sciences, National Research Centre, Dokki, Giza 12622, Egypt; 7 Center for Imaging and Microscopy (CIM), Zewail City of Science and Technology, October Gardens, 6th of October, Giza 12578, Egypt

**Keywords:** schiff base/ester, Lateral methoxy, optical properties, nematic stability, binary phase diagram

## Abstract

New mesomorphic series, 4-hexadecyloxy phenyl-imino-4′-(3-methoxyphenyl)-4″-alkoxybenzoates (**A***n*), were prepared and investigated with different thermal and mesomorphic techniques. The synthesized homologous series constitutes four members that differ from each other in the terminal length of flexible chain (*n*) attached to phenyl ester moiety, which varies between *n* = 6, 8, 10, and 12 carbons. A lateral CH_3_O group is attached to the central benzene ring in the meta position with respect to the ester moiety. Molecular structures of all newly prepared homologues were elucidated via FT-IR, ^1^H and ^13^C NMR spectroscopy. Mesomorphic and thermal properties were examined by differential scanning calorimetry (DSC), thermogravimetric analysis (TGA) and the mesophases identified by polarized optical microscopy (POM). DSC and POM examinations revealed that all members of the present series (**A***n*) exhibit a purely enantiotropic nematic (N) phase. Comparative evaluations and binary phase diagrams were established between the present homologues and their corresponding shorter one (**B***n*). The examination revealed that, the length of the flexible alkoxy chain incorporated into the phenylimino moiety is highly effective on the temperature range and stability of the mesophase observed. With respect to the binary mixtures **A***n*/**B***n*, the exhibited N phase showed to cover the whole composition range with eutectic behavior.

## 1. Introduction

The optical and mesomorphic characters of compounds are known to be mainly dependent on their architecture, in which a slight change in the molecular geometry is accompanied with considerable changes in their optical characteristics [1,2,3,4,5,6]. In order to understand the relationship between the molecular shape and the liquid crystalline (LC) properties is, in principle, a fundamental task in condensed material science, where it enables scientists to prepare LC materials with the desired properties. The design of new non-symmetrical compounds is associated with new molecular parameters. The first LC material prepared at room temperature, 4-methoxybenzylidene-4′-butylaniline (MBBA), was prepared by Kelker, H., et al. [7]. Several calamitic thermotropic LC Schiff base/ester LCs have been investigated and are often evaluated their interesting optical phenomena [8,9,10,11,12,13,14]. The geometrical studies indicated that each of the orientations of the ester within the rigid portion of the molecule, the location of azomethine and ester linking groups, laterally protruded groups, and the terminal flexible chain length, have important roles in the enhancement of the phase thermal stability. The rigid shape of the azomethine molecule makes them essential for exhibiting the mesomorphic phase with a high thermal stability [15,16,17]. Moreover, their interesting properties provide the possibility of molecular mobility in response to light or heat, thus offering many opportunities in photonic applications [18,19,20,21].

Generally, as the breadth of the molecule is increased, the thermal transitions of formed mesophases will be reduced [22]. In such cases, the inclusion of a compact polar lateral group to the main skeleton of the molecule influences the physical properties of the resulting LC material, such as the melting point, phase transition temperatures, morphology, dielectric anisotropy and dipole moment [23,24,25,26]. The small volume of lateral substituent enables its attachment into the mesomorphic shape of the molecule without being sterically disrupted, and accordingly, the LC mesophases can still be observed. On the other hand, the increased intermolecular separation, affected by the addition of the lateral group, broadens the core portion and leads to a reduction in the lateral interactions [27]. Furthermore, the longer flexible terminal chains in the molecules enhance their orientation in parallel alignments [28]. Actually, the lateral or terminal polar substituents induce the mesomeric properties of wide numbers of Schiff bases/ester derivatives [16,29]. The conformation of the alkyl terminal chain causes highly effects in the thermal and physical properties as the -CH2- and -CH3 numbers in the terminal chain changes from odd to even in LC mesophases; this effect is named the odd-even effect in a series [30]. Compounds with an odd number of C atoms in the terminal alkyl chain are more flexible than molecules with an even number of CH2 units. This behavior results in a less uniform orientation of LC materials [31]. Moreover, in the total chain length, odd and even numbers of C atoms result in a different macroscopic characteristics [31,32]. Thus, the odd-even effect offers a new mode to optimize mesophase and optical properties [33].

In order to dropping the melting temperature of a liquid crystalline material to be close to room temperature, the mixing of two or more components was established. The mixtures of LC show mesophase transitions and thermal properties that differ from their individual components. Mixing of two or more LC components leads to tuning the needed characteristics of interest [34,35,36,37,38,39,40,41,42]. Moreover, the binary LC mixtures could be better established for many fields in certain applications [43]. Thus, the mesomorphic characterization of such mixtures is of considerable interest [44,45,46]. These types of mixtures have been shown to achieve lower melting points [46]. Furthermore, the mesophase temperature range, observed for the eutectic mixture, is wider than either of its pure components [44,45,46,47].

**Scheme 1:**
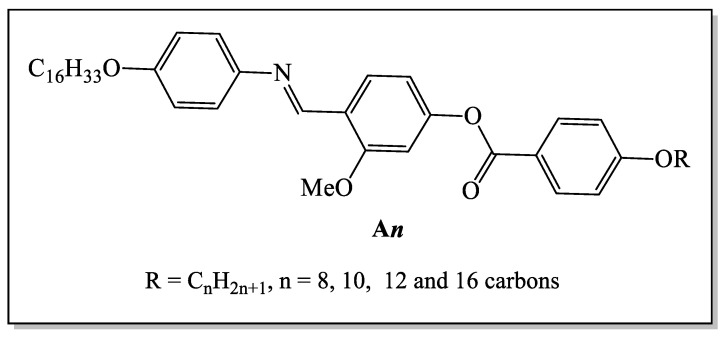
Prepared homologues series, A*n*.

In our previous work [48], the thermal and optical properties of 4-hexyloxyphenylimino-4′-(3-methoxyphenyl)-4″-alkoxy- benzoates were investigated and the results showed that all the homologues studied possess the nematic mesophase enantiotropically. Herein, the aim of present work is to synthesize a homologous series; namely, 4-hexdecyloxyphenylimino-4′-(3-methoxyphenyl)-4″-alkoxy- benzoates, **A***n*, of long terminal alkyl chain length connected to the end of phenyl azomethine linkage, with different alkoxy terminal groups of different lengths, on the other end of the molecule (Scheme 1). The study will be extended to investigate their mesomorphic behavior and the effect of the terminal length of alkoxy chain (n) on their mesomorphic phenomena. Finally, binary mixtures of different azomethine homologues bearing different terminal chain lengths were investigated in order to evaluate the mesophase behavior in their mixed states.

## 2. Experimental

### 2.1. Synthesis

**Scheme 2:**
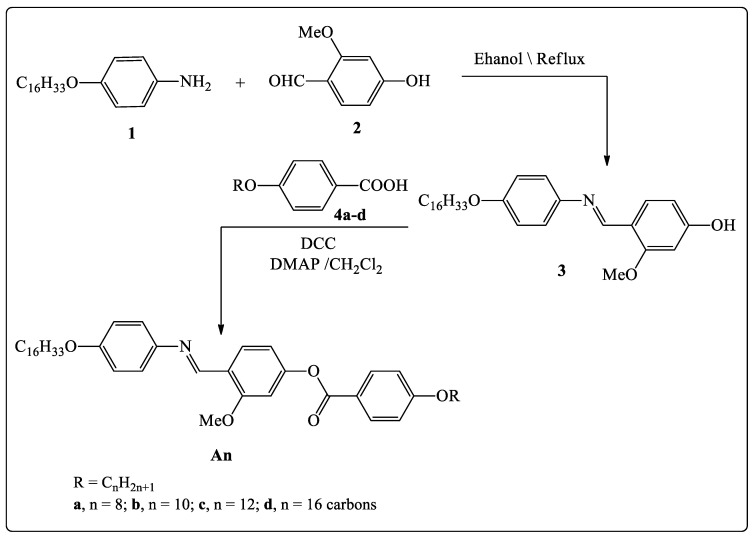
Synthesis route of title compounds **A***n*.

Schiff base and hydrazone derivatives are well known as valuable intermediates in the synthesis of many organic compounds that are used in a multitude of applications [49,50,51,52,53,54,55,56,57]. A series of new laterally methoxy Schiff base derivatives **A***n* were prepared according to the following Scheme 2:

#### 2.1.1. Synthesis of 4-((4-(Hexadecyloxy)Phenyliminomethyl)-3-Methoxyphenol (3)

Details were inserted in Supplementary Data.

#### 2.1.2. Synthesis of 4-((4-(Hexadecyloxy)Phenyl)Imino M thyl)-3-Methoxyphenyl4-Alkoxybenzoate, **A***n*

Details were inserted in Supplementary Data.

Elemental analyses, Infrared spectra (FT-IR) and ^1^H-NMR, for the designed series were agreement with the structures assigned. ^1^H-NMR results displayed the expected integrated aliphatic to aromatic proton ratios in all prepared compounds.

## 3. Results and Discussion

### 3.1. Mesomorphic Behavior of Investigated Series, ***A***n

**Figure 1:**
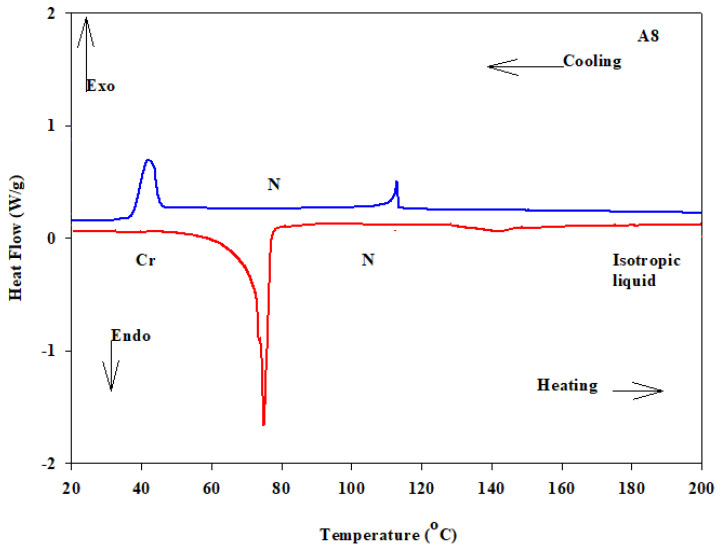
DSC thermograms of homologue **A***8* recorded from the second heating and cooling cycles at a rate of ±10 °C min^−1^.

**Figure 2:**
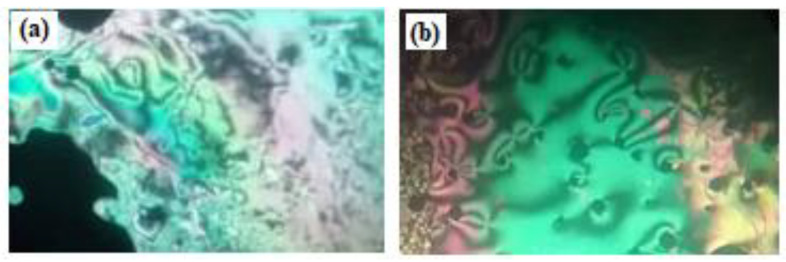
N mesophase textures observed upon heating under POM for (**a**) homologue **A***8* at 107.0 °C and (**b**) homologue **A***16* at 102.0 °C.

**Figure 3:**
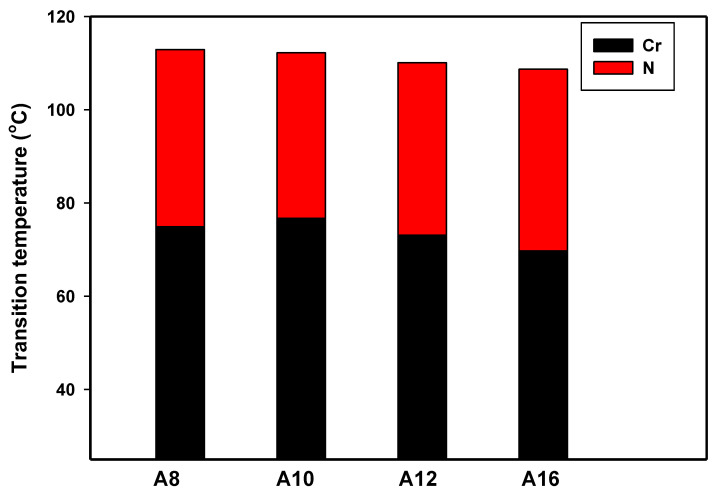
Impact of alkoxy chain length (*n*) on the mesomorphic behavior of the present investigated series, **A***n*.

The mesophase transition temperatures and optical behaviors of the investigated laterally methoxy homologous series were evaluated by DSC and POM measurements. A DSC thermogram of the homologue **A***8* is displayed in Figure 1 upon heating and cooling cycles, as a representative example. Two transition peaks were observed in Figure 1. The nematic mesophase was observed either from Cr→ N on heating, or from I→ N on cooling. The position of the endothermic and exothermic peaks observed depended on the length of the attached terminal alkoxy chain. The endothermic peaks were ascribed to mesophase transition upon heating and the exothermic peaks upon cooling. Optical images under POM confirm the DSC measurements. Figure 2 illustrates the POM nematic phase texture of the **A***8* homologue, as an example. The phase transition temperatures, as driven from measurements via DSC, and their associated enthalpies of transitions for all of the homologues series, **A***n*, are collected in Table 1. In order to investigate the effect of the length of the terminal alkoxy chain (*n*) on the mesomorphic properties of prepared compounds, Figure 3 displays their relationship. As shown from Table 1 and Figure 3, all the synthesized homologues are mesomorphic in nature with enantiotropic properties and their thermal mesophase stability (the maximum temperature stability of the phase) is dependent on their terminal flexible alkoxy-chain length (*n*). Moreover, all were shown to possess a pure nematic phase. It can also be seen from Table 1 and Figure 3 that the melting temperature of compounds varied as usual randomly with *n*. The shortest terminal length member (**A***8*) exhibits an N phase with thermal stability (***T***_N-I_) and a temperature range (**Δ*T***) of 112.9 and 38.0 °C, respectively. For **A***10*, it possesses also the enantiotropic N mesophase with nematogenic stability and ranges that are nearly 112.2 and 35.5 °C, respectively. The homologue **A***12* possesses less thermal N stability (110.1 °C) and the N mesophase temperature range is nearly 37.0 °C. The longest derivative A*16* exhibits the lowest thermal N stability (108.7 °C) and the highest nematogenic temperature range nearly 39.0 °C. In general, the shape of the molecule, polarizability and the dipole moment of the designed molecule are highly impacted by the electronic nature of the terminal substituents. Moreover, the mesomorphic behavior is influenced by an increment in the polarity and/or polarizability of the molecular mesogenic cores. The nematogenic range of the present investigated homologues series decreases in the order: **A***16* > **A***8* > **A***12* > **A***10*. Mesomorphic phenomena indicate the sharing of these factors with different extents. The aggregation due to the oxygen of the alkoxy chain and the ester carbonyl moiety and the cohesive forces between molecules are important factors that determine the observed nematic phase [58,59].

The normalized entropy changes (ΔS_N-I_/R) of the prepared homologous series are summarized in Table 1. The results revealed that the value of the entropy changes is observed to be related, independently, to the chains terminal length (*n*). The small values observed for the entropy change can be attributed to the decrease in the ratio of length/breadth as a result of their lower anisotropy due to their molecular shape and molecular biaxiality [60,61]. The induction, conjugation forces, the specific dipolar interactions, as well as the π-π stacking interactions, play essential roles in the molecular orientation and thus in the arrangement of molecules, as well as in the formation of the mesophase. Moreover, the thermal cis/trans isomerization of the –CH=N-linkage was an essential factor in the observed entropy change values. The higher entropy changes of the **A***10* derivative may be attributed to the increment of its molecular biaxiality in addition to differences in its molecular interactions.

### 3.2. Thermal Characterizations

**Figure 4:**
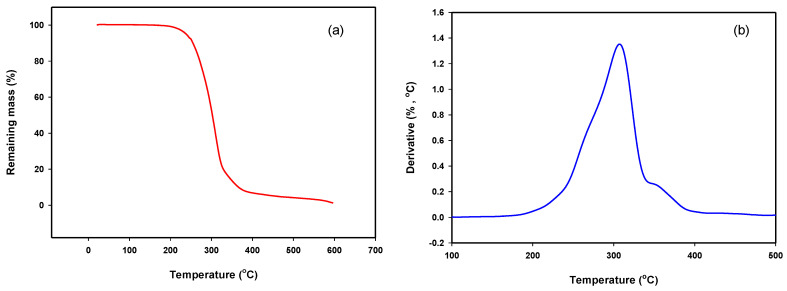
TG (**a**) and DTG (**b**) curves of the compound **A***8*.

The thermal stabilities of the prepared compounds (**A***n*) were evaluated by thermogravimetric analysis (TG). The TG thermogram and its corresponding derivative (DTG) of the homologue **A***8* is displayed in Figure 4, as a representative example. As can be seen from Figure 4, the decomposition takes place through two degradation steps depending on the molecular structure of the compound. The first step occurs in the temperature range ≈ 220–310 °C and starts at 240 °C with maximum degradation rate (**T**_max_) at 310 °C, indicating that the sample has a high thermal stability, while the second decomposition step occurs between 320 °C and 380 °C with maximum degradation rate ≈ 340 °C. The results revealed that the investigated materials possess high thermal stabilities of up to 340 °C, which covers the temperature window of phase transitions.

### 3.3. Effect of the Proportionating of the Alkoxy Chain Length on the Mesomorphic Properties

**Scheme 3:**
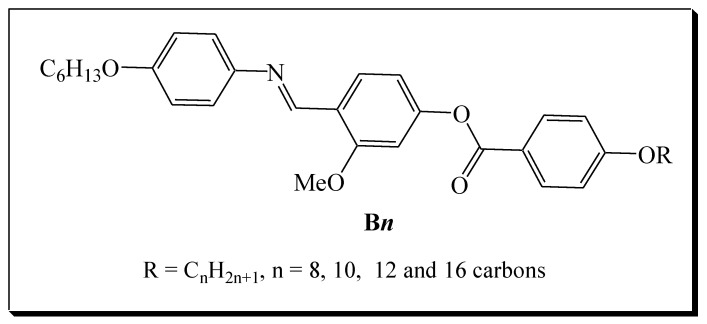
Homologues series, **B***n*.

**Figure 5:**
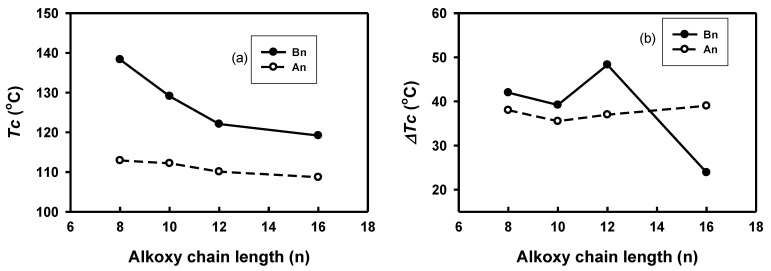
Comparison of **T**c (**a**); and Δ**T**c (**b**) of series **A***n* and **B***n*.

The terminal alkoxy chain attached to the molecule has an essential role to impact their mesomorphic transitions. In order to analyze the impact of the proportion of the alkoxy chain length, which is connected to the terminal azomethine phenyl moiety, on the mesomorphic phenomena of the series, a comparison was made between the phase behavior of the present long-chain analogues (**A***n*) and the previously short chain homologues series **B***n* [48] (Scheme 3) and the correlation is graphically depicted in Figure 5a and their temperature ranges in Figure 5b. As can be seen from Figure 5a, the mesomorphic thermal stability of **B***n* homologues are higher than those of **A***n*. This may be attributed to the influenced mesogenic core dipole moments, which is dependent upon the mesomeric nature of the short chain (CH_2_ = 6). The mesomorphic range (Figure 5b) of the **B***n* series exhibits higher temperature ranges than those of **A***n***,** except for *n* = 16 carbons. The present homologous series **A***n* showed a wider nematic temperature range than the previously investigated analogues, **B***n*. The constructed comparison revealed that the nematic thermal stability, as well as its temperature range, depends also on the length of the alkoxy chain attached to the phenylimino moiety. The flexibility of the terminal alkoxy chains and the rigidity of phenyl rings in the central backbone are important in the LC phase formation. Moreover, the semi-flexibility of terminals has a big role in the thermal stability of LC phases. That is, as the terminal alkoxy chain length of the molecule increases, the rigidity of the central part will be decreased; so, the linearity of the molecule slightly decreases due to the greater number of configurations of the terminal chains (which lead to the strong terminal interactions).

### 3.4. Binary Phase Diagrams of Components of Different Terminal Alkoxy Chain Lengths

The binary mixture phase diagrams are changed depending on the kind of its individual components. The different polarity, as well as the location of the lateral substituent, is the main factors. The goal of such a study was to attain a balance between the two components, which are expected to be improved on mixing, thus resulting in advantageous physical and thermal properties, as well as depression of the melting transitions.

**Figure 6:**
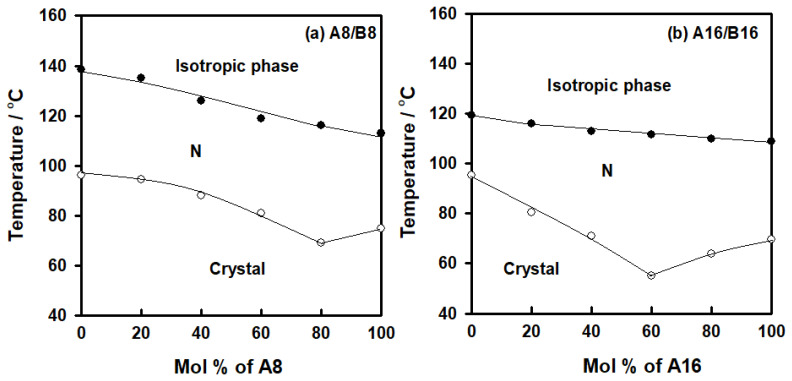
Phase diagrams for systems (**a**) **A8/B8** and (**b**) **A16/B16**; the symbol “o” denotes the solid–N phase, “●” N–I phase transition upon second heating scan.

**Figure 7:**
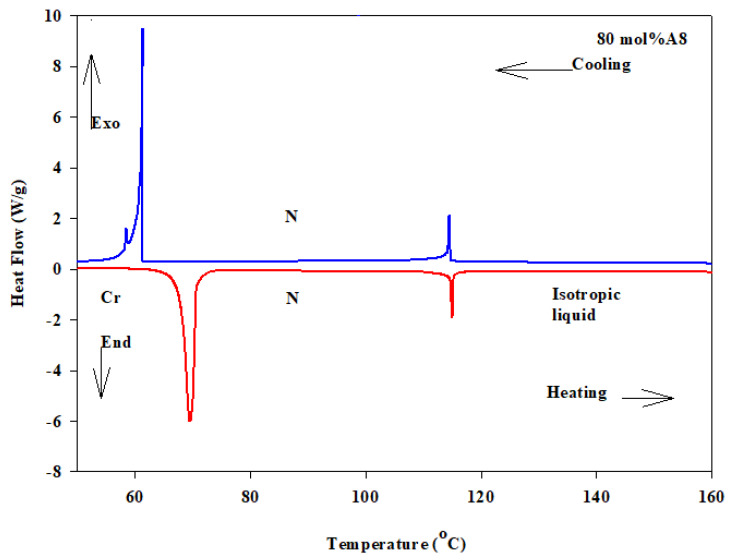
DSC curve of 80% mol of **A***8* for system **A8/B8** at a rate of ±10 °C min^−1^ recorded upon heating and cooling cycles.

The constructed phase diagrams, derived from measured DSC curves of mixtures, are made from different homologues with different alkoxy chain-lengths and are depicted in Figure 6a,b. As can be seen from Figure 6a,b, the mixtures constructed from compounds bearing short alkoxy-chain groups (**A8/B8**) and the longest terminal chains (**A16/B16**) were shown to possess the N phase over the entire composition range. Slight decrements from the ideal behavior are observed for the clearing temperatures of both systems. This may be due to the molecular association disruption, as a result of the dis-similarity of the alkoxy terminal chain lengths, in the two components as is observed in the previously reported binary systems [62]. In both systems, their solid mixtures have eutectic melting temperatures of 69.1 °C and 55.3 °C with eutectic compositions 79.9 and 59.6 mol% for **A8/B8** and **A16/B16**, respectively. On the other hand, the eutectic mixture of binary system **A8/B8** exhibits an N temperature range of nearly 47.0 °C, while the N range for the eutectic mixture of the **A16/B16** system is about 56.3 °C. It can be concluded that the two mixed analogues **A***n* and **B***n* have different aspect ratios, in which one derivative is longer than the other, which may be located in the organized directed geometry; so, the addition of the designed derivatives of **A***n* are not expected to disturb the molecular arrangement of the shorter homologues **B***n*. Typical DSC curve of heating and cooling scans of the mixture **A8/B8** of composition 80% mol of **A***8* (at eutectic composition) are displayed in Figure 7.

## 4. Conclusions

A new homologue set based on the laterally-methoxy substituted azomethine derivatives was synthesized and investigated with different optical and thermal techniques. Molecular structures were confirmed by FT-IR and NMR spectroscopy. The optical, mesomorphic and thermal properties were determined using DSC, POM, and TGA analyses. The results revealed that all synthesized compounds exhibit only an N phase irrespective of their terminal alkoxy-chain length. Moreover, their thermal stability was examined and shown to cover a higher stability than their mesomorphic transitions. A comparative investigation was made between the present designed series and their corresponding short-chain homologues. The study revealed that the attached flexible chain to the phenylimino portion is more effective on the observed nematic temperature range and stability. The binary phase mixtures of **A***n*/**B***n* showed an enantiotropic N phase covering the whole composition range, with depression in the melting temperature at the eutectic composition.

## Figures and Tables

**Table 1:** Mesomorphic transition temperatures (°C), enthalpy (**ΔH**) of transition, kJ/mole, and normalized entropy (**ΔS**/R) of transition for homologues **A***n*.

Comp.	*T*_Cr-N_	Δ*H*_Cr-N_	*T*_N-I_	Δ*H*_N-I_	Δ*T*	Δ*S*_N-I_/R
**A** *8*	74.9	62.39	112.9	1.59	38.0	0.50
**A** *10*	76.7	48.4	112.2	2.70	35.5	0.84
**A** *12*	73.1	40.90	110.1	1.56	37.0	0.49
**A** *16*	69.7	44.65	108.7	1.63	39.0	0.51

Cr-N = transition of solid to the nematic phase. N-I = transition of nematic to the isotropic liquid phase. **ΔH** = Enthalpy of transition, kJ/mole derived from DSC peak of transitions; **ΔS**/R = **ΔH**/RT is the normalized entropy of transition, unitless (due to the entropy change **ΔS** is divided by **R** = gas constant).

## Data Availability

The data presented in this study are available on request from the corresponding author.

## Supplementary Materials

The following are available online at https://www.mdpi.com/article/10.3390/polym13111687/s1. The synthetic and characterization details of investigated compounds.
