# Supplementary material for: Optical and Thermal Investigations of New Schiff Base/Ester Systems in Pure and Mixed States
Source: Polymers (Basel). 2021 May 22;13(11):1687. doi: 10.3390/polym13111687 (PMC8196809; doi:10.3390/polym13111687)
Supplement: Supplementary file 1 [file polymers-13-01687-s001.zip › polymers-1239155-supplementary.pdf]

Supplementary data

Optical and thermal investigations of New Schiff base/ester Systems in pure and mixed states

Abeer S. Altowyan[1], Hoda A. Ahmed[2,3]*, Sobhi M. Gomha[2,4] and Ayman M. Mostafa[5,6,7]

[1]Department of physics, College of Science, Princess Nourah bint Abdulrahman University, Riyadh, Saudi Arabia.
[2]Department of Chemistry, Faculty of Science, Cairo University, Cairo 12613, Egypt, ahoda@sci.cu.edu.eg, sm.gomha@iu.edu.sa
[3]Chemistry Department, College of Sciences, Yanbu, Taibah University, Yanbu 30799, Saudi Arabia.
[4]Chemistry Department, Faculty of Science, Islamic University in Almadinah Almonawara, Almadinah Almonawara, 42351, Saudi Arabia.
[5] Spectroscopy Department, Physics Division, National Research Centre, El-Buhouth St., Dokki, Giza, Egypt
[6]Laser Technology Unit, Centre of Excellence for Advanced Sciences, National Research Centre, Dokki, Giza, Egypt
[7]Center for Imaging and Microscopy (CIM), Zewail City of Science and Technology, October Gardens, 6th of October, Giza, 12578, Egypt

** Correspondence: H. A. Ahmed, ahoda@sci.cu.edu.eg*

---

1. *Materials*

4-Alkyloxybenzoic acids (*n = 8, 10, 12, and 16 carbons)*, vaniline, and 4-(hexadecyloxy) aniline, were purchased from Sigma Aldrich (Germany). dichloromethane, *N,N'*-dicyclohexylcarbodiimide (DCC), ethanol and 4-dimethylaminopyridine (DMAP) were purchased from Aldrich (Wisconsin, USA).

2. *Synthesis of* 4-((4-(hexadecyloxy)phenyliminomethyl)-3-methoxyphenol (3)

A mixture of 4–hydroxy-2-methoxybenzaldehyde (1.52g, 10 mmol) and 4-(hexadecyloxy)aniline (3.33g, 10 mmol) in ethanol (20 mL) were refluxed for two hours (monitored by TLC). The mixture was then cooled to room temperature and filtered. The solid obtained was washed with cold ethanol and recrystallized twice from hot ethanol to give pure imine compound **3** as indicated by TLC technique.

The melting points and IR data determined for the prepared imine **3:** Yield: 88.9%; mp 86.5 °C, FTIR (ύ, cm^{-1}): 3413 (OH), 2993, 2924 (CH_2 stretching), 1731 (C=O), 1612 (C=N), 1573 (C=C), 1471 (C–O_{Asym}), 1259 (C-O_{Sym}). ^{1}H-NMR (400 MHz, $CDCl_3$): *δ*/ppm: 0.92 (t, 3H, $\underline{CH_3}(CH_2)_{13}CH_2CH_2O$-), 1.24-1.41 (m, 26H, $CH_3\underline{(CH_2)_{13}}CH_2CH_2O$-), 1.69-175 (q, 2H, $CH_3(CH_2)_{13}\underline{CH_2}CH_2O$-), 3.73 (s, 1H, OH), 3.90 (s, 3H, $O\underline{CH_3}$), 3.97-4.05 (t, 2H, $CH_3(CH_2)_{13}CH_2\underline{CH_2}O$-), 6.82 (d, 2H, Ar–H), 6.91 (d, 2H, Ar–H), 7.03 (d, 2H, Ar–H), 7.13 (d, 2H, Ar–H), 7.25 (s, 1H, Ar–H), 7.36 (d, 2H, Ar–H), 9.73 (s, 1H, CH=N). Anal. Calcd. for $C_{30}H_{45}NO_3$ (467.68): C, 77.04; H, 9.70; N, 2.99. Found: C, 77.16; H, 9.55; N, 2.79%.

3. General procedure for synthesis of 4-((4-(hexadecyloxy)phenyl)imino methyl)-3-methoxyphenyl4-alkoxybenzoate, A*n*

A mixture of imine compound **3** (4.67g, 10 mmol) and the appropriate 4-alkoxy benzoic acids (10 mmol for each) in dry methylene chloride (25 mL) containing *N*, *N'*–dicyclohexylcarbodiimide (DCC, 10 mmol) and few crystals of 4-dimethylaminopyridine (DMAP), as catalyst, were left to stand for 72 hours at room temperature with continuous stirring. The solid byproduct separated was then filtered off and the solution evaporated. The solid remained after evaporation was recrystallized from ethanol to give TLC pure products. The purity of the prepared samples was checked with thin-layer chromatography (TLC) using TLC sheets coated with silica gel (E Merck), and CH_2Cl_2/CH_3OH (9:1) as eluent, whereby only one spot was detected by a UV-lamp.

The physical data of products **A***n* are listed below:

4-(4-(Hexadecyloxyphenyl)iminomethyl)-3-methoxyphenyl-4-octyloxybenzoate (A*8*).

Yield: 93.5%; mp 75.0°C, FTIR (ύ, cm^{-1}): 2940, 2862 (CH_2 stretching), 1729 (C=O), 1609 (C=N), 1536 (C=C), 1461 (C–O_{Asym}), 1241 (C-O_{Sym}). ^{1}H-NMR (400 MHz, $CDCl_3$): *δ*/ppm: 0.85-0.90 (m, 6H, $\underline{CH_3}(CH_2)_{13}CH_2CH_2O$- and $\underline{CH_3}(CH_2)_5CH_2CH_2O$-), 1.25-1.32 (m, 36H, $CH_3\underline{(CH_2)}_{13}CH_2CH_2O$- and $CH_3\underline{(CH_2)}_5CH_2CH_2O$-), 1.72-1.78 (m, 4H, $CH_3(CH_2)_{13}\underline{CH_2}CH_2O$- and $CH_3\underline{(CH_2)}_5CH_2CH_2O$-), 3.86 (s, 3H, $O\underline{CH_3}$), 3.88-3.91 (t, 2H, $CH_3(CH_2)_{13}CH_2\underline{CH_2}O$-), 4.01-4.04 (t, 2H, $CH_3(CH_2)_5CH_2\underline{CH_2}O$-), 6.78-6.98 (m, 4H, Ar–H), 7.24-7.32 (d, 2H, Ar–H), 7.49-7.51 (d, 2H, Ar–H), 8.10-8.13 (m, 3H, Ar–H), 9.95 (s, 1H, CH=N) ppm. Anal. Calcd. for $C_{45}H_{65}NO_5$ (700.00): C, 77.21; H, 9.36; N, 2.00. Found: C, 77.08; H, 9.27; N, 1.83%.

4-(4-(Hexadecyloxyphenyl)iminomethyl)-3-methoxyphenyl-4-decyloxybenzoate (A*10*)

Yield: 91.0%; mp 77.0 °C, FTIR (ύ, cm^{-1}): 2967, 2838 (CH_2 stretching), 1732 (C=O), 1605 (C=N), 1572 (C=C), 1466 (C-O_{Asym}), 1265 (C-O_{Sym}). ^{1}H-NMR (400 MHz, $CDCl_3$): δ/ppm: 0.82-0.91 (m, 6H, $\underline{CH_3}(CH_2)_{13}CH_2CH_2O$- and $\underline{CH_3}(CH_2)_7CH_2CH_2O$-), 1.243-1.37 (m, 40H, $CH_3\underline{(CH_2)}_{13}CH_2CH_2O$- and $CH_3\underline{(CH_2)}_7CH_2CH_2O$-), 1.74-1.80 (m, 4H, $CH_3(CH_2)_{13}\underline{CH_2}CH_2O$- and $CH_3\underline{(CH_2)}_7CH_2CH_2O$-), 3.90 (s, 3H, $O\underline{CH_3}$), 3.97-4.03 (m, 4H, $CH_3(CH_2)_{13}CH_2\underline{CH_2}O$- and $CH_3(CH_2)_7CH_2\underline{CH_2}O$-), 6.87-6.99 (m, 4H, Ar–H), 7.30-7.34 (d, 2H, Ar–H), 7.43-7.52 (d, 2H, Ar–H), 8.03-8.16 (m, 3H, Ar–H), 9.91 (s, 1H, CH=N) ppm. Anal. Calcd. for $C_{47}H_{69}NO_5$ (727.52): C, 77.54; H, 9.55; N, 1.92. Found: C, 77.36; H, 9.63; N, 1.81%.

4-(4-(Hexadecyloxy)phenyl)iminomethyl)-3-methoxyphenyl-4-dodecyloxy benzoate (A*12*)

Yield: 92.5%; mp 73.0 °C, FTIR (ύ, cm^{-1}): 2963, 2889 (CH_2 stretching), 1730 (C=O), 1606 (C=N), 1575 (C=C), 1455 (C-O_{Asym}), 1473 (C-O_{Asym}). ^{1}H-NMR (400 MHz, $CDCl_3$): δ/ppm: 0.79-0.85 (m, 6H, $\underline{CH_3}(CH_2)_{13}CH_2CH_2O$- and $\underline{CH_3}(CH_2)_9CH_2CH_2O$-), 1.20-1.42 (m, 44H, $CH_3\underline{(CH_2)}_{13}CH_2CH_2O$- and $CH_3\underline{(CH_2)}_9CH_2CH_2O$-), 1.64-1.79 (m, 4H, $CH_3(CH_2)_{13}\underline{CH_2}CH_2O$- and $CH_3\underline{(CH_2)}_9CH_2CH_2O$-), 3.85 (s, 3H, $O\underline{CH_3}$), 3.89-4.06 (t, 4H, $CH_3(CH_2)_{13}CH_2\underline{CH_2}O$- and $CH_3(CH_2)_9CH_2\underline{CH_2}O$-), 6.73-6.89 (m, 4H, Ar–H), 7.19-7.39 (m, 4H, Ar–H), 7.98-8.03 (m, 3H, Ar–H), 9.83 (s, 1H, CH=N) ppm. Anal. Calcd. for $C_{49}H_{73}NO_5$ (755.55): C, 77.84; H, 9.73; N, 1.85. Found: C, 77.70; H, 9.64; N, 1.77%.

4-(4-(Hexadecyloxy)phenyl)iminomethyl)-3-methoxyphenyl-4-hexadecyloxy benzoate (A1*6*)

Yield: 94.5%; mp 70.0 °C, FTIR (ύ, cm^{-1}): 2957, 2871 (CH_2 stretching), 1731 (C=O), 1607 (C=N), 1551 (C=C), 1473 (C-O_{Asym}), 1254 (C-O_{Sym}). ^{1}H-NMR (400 MHz, $CDCl_3$): δ/ppm: 0.84-0.90 (m, 6H, 2x$\underline{CH_3}(CH_2)_{13}CH_2CH_2O$-), 1.24-1.31 (m, 52H, 2x$CH_3\underline{(CH_2)}_{13}CH_2CH_2O$-), 1.72-1.82 (m, 4H, 2x$CH_3(CH_2)_{13}\underline{CH_2}CH_2O$-), 3.86 (s, 3H, $O\underline{CH_3}$), 4.00--4.04 (m, 4H, 2x$CH_3(CH_2)_{13}CH_2\underline{CH_2}O$-), 6.78-6.98 (m, 4H, Ar–H), 7.24-7.32 (d, 2H, Ar–H), 7.48-7.51 (d, 2H, Ar–H), 8.11-8.13 (m, 3H, Ar–H), 9.95 (s, 1H, CH=N) ppm. Anal. Calcd. for $C_{53}H_{81}NO_5$ (811.61): C, 78.37; H, 10.05; N, 1.72. Found: C, 78.24; H, 10.00; N, 1.61%.

4. Binary mixture preparations

Binary mixtures were prepared by mixing accurately weighed samples of the appropriate components (±1.0 % in composition), melting them together to give an intimate mixture, and then cooled to room temperature with stirring. For the construction of the binary phase diagram, the mixtures of any two components were made to cover the whole range of composition.

5. *Characterization*

Perkin-Elmer B25 (Perkin-Elmer, Inc., Shelton, CT USA) spectrophotometer was used for infrared spectra measurements. Varian EM 350L 500 MHz spectrometer (Oxford, UK) was used for recording ^{1}HNMR spectra with tetramethyl silane as internal standard in $CDCl_3$; the chemical shift values were recorded as δ (ppm units). Thermo Scientific Flash 2000 CHS/O Elemental Analyzer, Milan, Italy was used for Elemental analyses.

TA Instruments Co. (Q20 Differential Scanning Calorimeter, DSC; USA) was used for recording phase transitions. DSC calibration was carried out using lead and indium melting temperature and enthalpy, as references. Samples of 2–3 mg were used in aluminum pans for DSC investigation. The heating rate was 10°C/min in nitrogen gas as an inert atmosphere (30 ml/min). All transitions measured form the second heating scan.

Thermogravemetric analysis (TGA) was carried out using Shimadzu TGA-50H Thermal Analyzer under nitrogen at a heating rate of 10ºC/min. The measurements were carried out from room temperature up to 600ºC, and the reference material was α-alumina. The sample weights for all the experiments were taken in the range of 3–4 mg.

Transition temperatures for the prepared homologues were checked and phases identified by Polarized optical microscope (POM, Wild, Germany) attached with Mettler FP82HT hot stage.

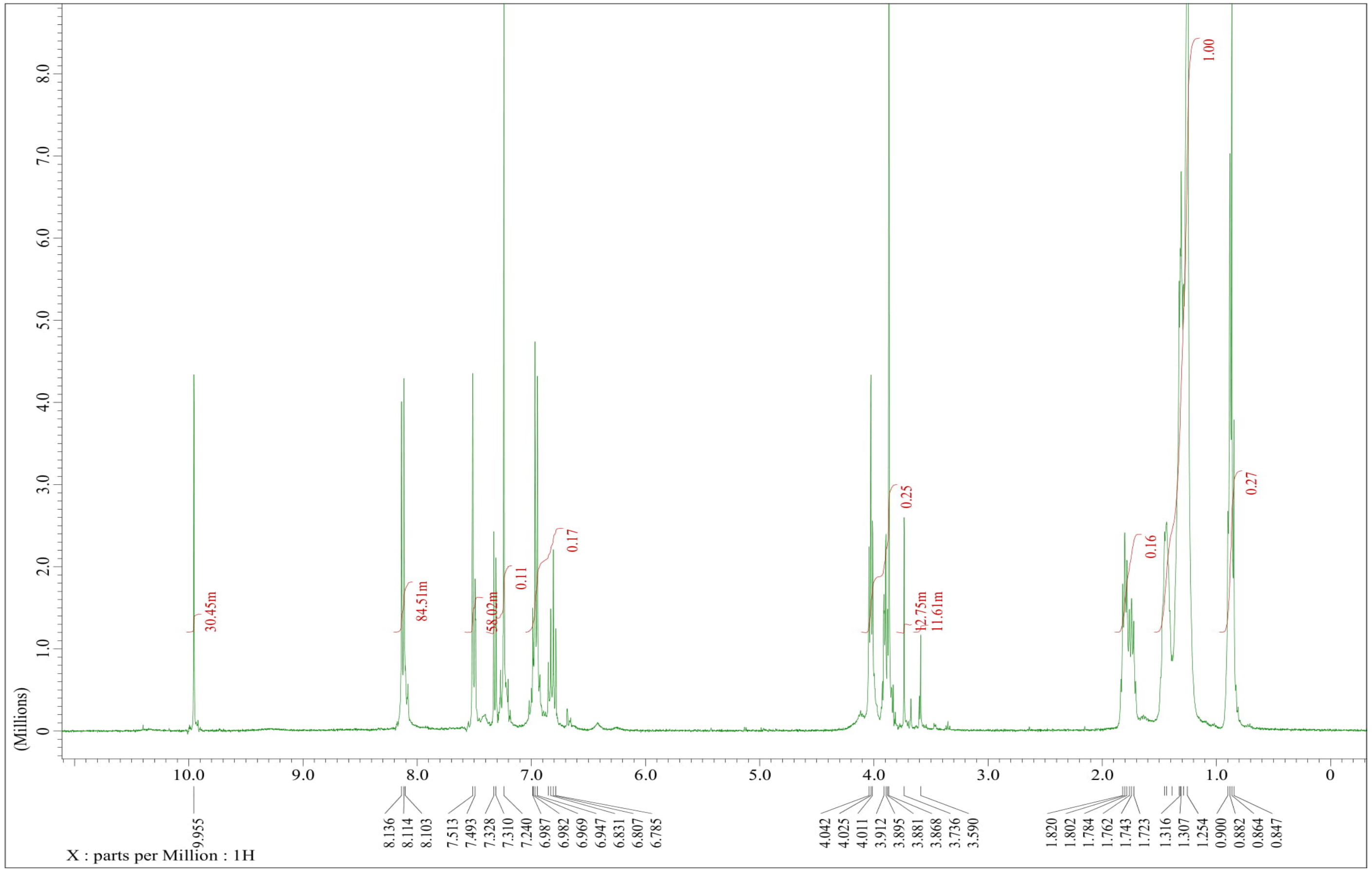

Figure S1: ¹H-NMR spectra of compound A*8*

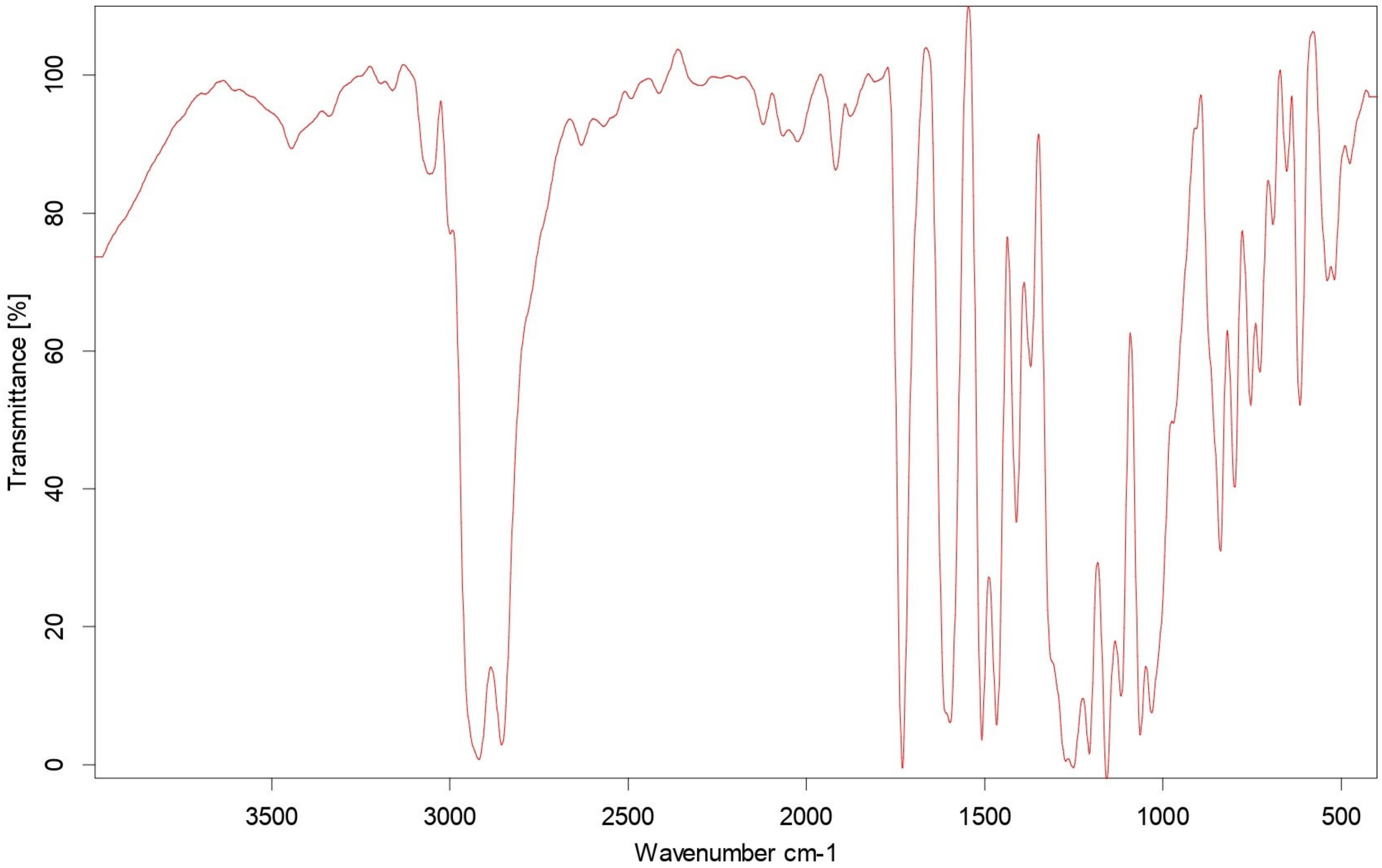

Figure S2: FT-IR spectra of compound A*8*

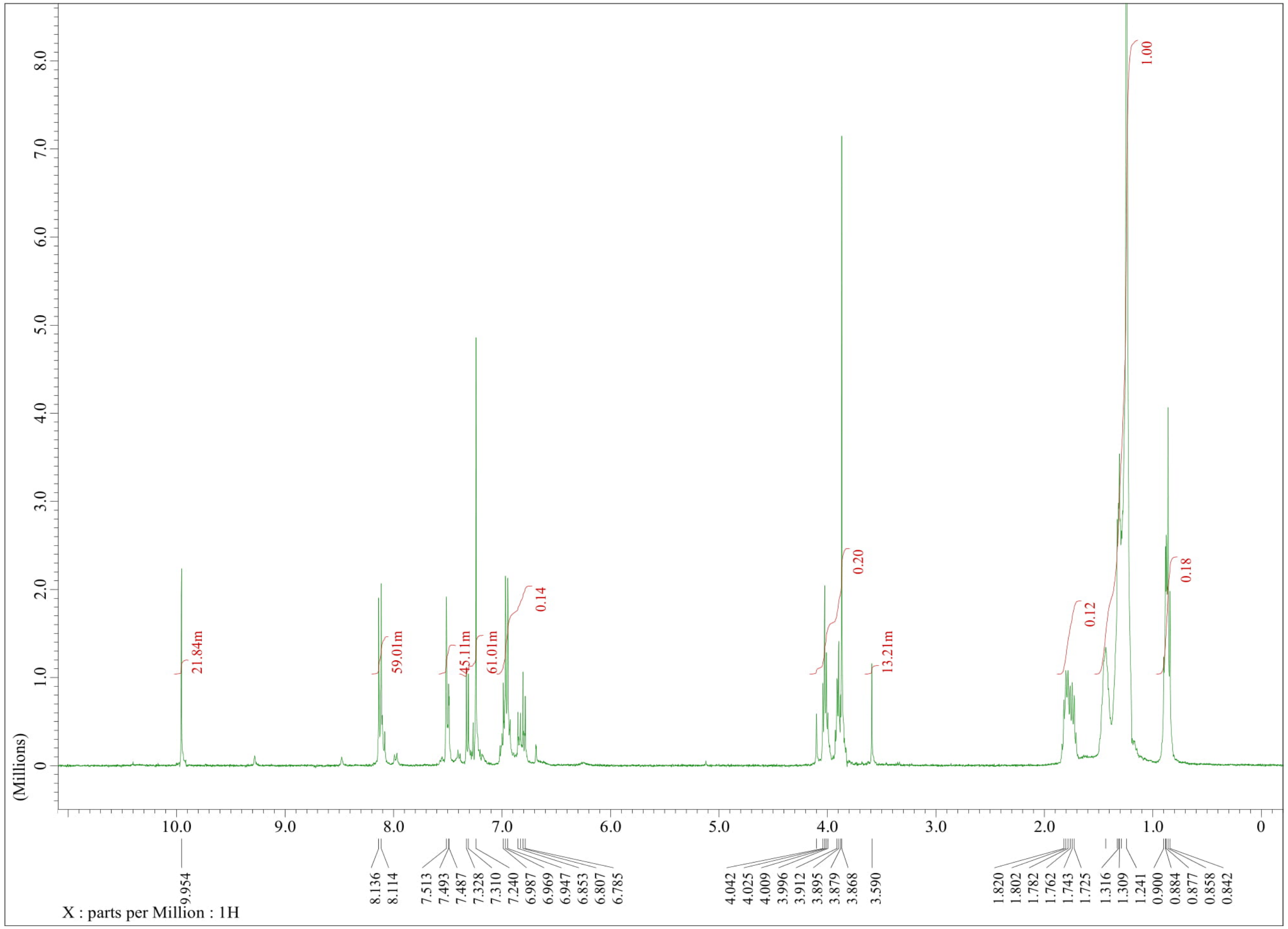

Figure S3: ^{1}H-NMR spectra of compound A*16*

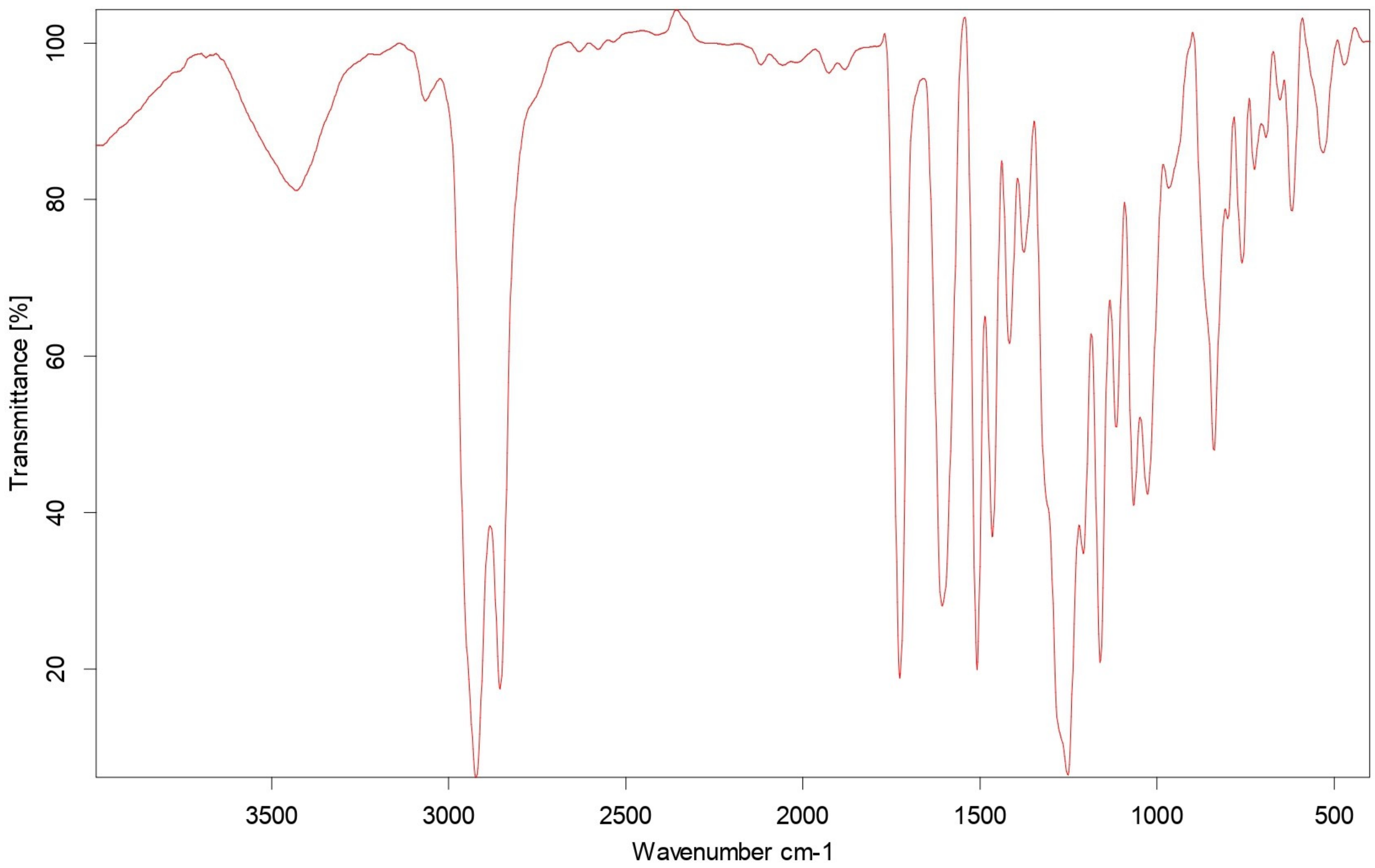

Figure S4: FT-IR spectra of compound A*16*
